# Psilocybin-induced changes in neural reactivity to alcohol and emotional cues in patients with alcohol use disorder: an fMRI pilot study

**DOI:** 10.1038/s41598-024-52967-8

**Published:** 2024-02-07

**Authors:** B. A. Pagni, P. D. Petridis, S. K. Podrebarac, J. Grinband, E. D. Claus, M. P. Bogenschutz

**Affiliations:** 1 Department of Psychiatry, NYU Langone Center for Psychedelic Medicine, NYU Grossman School of Medicine, New York, NY USA; 2 Departments of Psychiatry and Radiology, Columbia University Vagelos College of Physicians & Surgeons, New York, NY USA; 3 Department of Biobehavioral Health, The Pennsylvania State University, University Park, PA USA

**Keywords:** Randomized controlled trials, Neural circuits, Functional magnetic resonance imaging

## Abstract

This pilot study investigated psilocybin-induced changes in neural reactivity to alcohol and emotional cues in patients with alcohol use disorder (AUD). Participants were recruited from a phase II, randomized, double-blind, placebo-controlled clinical trial investigating psilocybin-assisted therapy (PAT) for the treatment of AUD (NCT02061293). Eleven adult patients completed task-based blood oxygen dependent functional magnetic resonance imaging (fMRI) approximately 3 days before and 2 days after receiving 25 mg of psilocybin (n = 5) or 50 mg of diphenhydramine (n = 6). Visual alcohol and emotionally valanced (positive, negative, or neutral) stimuli were presented in block design. Across both alcohol and emotional cues, psilocybin increased activity in the medial and lateral prefrontal cortex (PFC) and left caudate, and decreased activity in the insular, motor, temporal, parietal, and occipital cortices, and cerebellum. Unique to negative cues, psilocybin increased supramarginal gyrus activity; unique to positive cues, psilocybin increased right hippocampus activity and decreased left hippocampus activity. Greater PFC and caudate engagement and concomitant insula, motor, and cerebellar disengagement suggests enhanced goal-directed action, improved emotional regulation, and diminished craving. The robust changes in brain activity observed in this pilot study warrant larger neuroimaging studies to elucidate neural mechanisms of PAT.

**Trial registration**: NCT02061293.

## Introduction

Alcohol use disorder (AUD) is a chronic relapsing condition characterized by an impaired ability to regulate or abstain from alcohol despite negative personal, occupational, and social consequences^1^. A three-domain model has been proposed to account for the core neuropsychological features of AUD^2^. The three domains are: (1) negative emotionality, which includes feelings of dysphoria, hypohedonia, hypersensitivity to stress, and withdrawal symptoms; (2) changes to incentive salience, including craving, reward habit formation, and attentional biases; (3) change to executive functioning, including goal-directed behavior, response inhibition, and cognitive flexibility^3^. These domains are broadly governed by amygdala/mPFC, striatum/insula, and lateral PFC functioning, respectively^4,5^. Further, neural responses to alcohol cues overlap with those identified in incentive salience (attentional biases) and negative emotionality paradigms^6,7^, suggesting that a shared neurobiological circuitry underpins deficits across domains. Of clinical importance, environmental (i.e., alcohol cues) and internal (i.e., stress and negative affect) cue-elicited craving are significant predictors of relapse and therefore provide a theoretical basis for probing neurobiological abnormalities in pursuit of novel treatment targets^8,9^.

Although medications exist for AUD, the effect sizes of currently approved treatments are disappointingly small and limited to particular sub-populations^10–12^. Furthermore, very few people with AUD are currently receiving treatment (only 1.6% in the US as of 2019^13^), which may be partially attributable to the ineffectiveness and side effect profiles of currently available medications^14^. However, emerging evidence suggests that psilocybin, the psychoactive constituent of magic mushrooms, may precipitate sustained reductions in drinking behavior after one or two drug administrations when paired with therapy with few significant side effects^15^. A Phase II randomized, placebo-controlled trial of psilocybin in patients with AUD demonstrated significant reductions in percent heavy drinking days, percent drinking days, and drinks per day 8 months post-treatment^16^. Relative to placebo, participants receiving psilocybin were more likely to report abstinence, no heavy drinking, and greater reductions in risky drinking after treatment.

Psilocybin is a nonspecific serotonin agonist that produces profound alterations in sensory, emotional, and cognitive perception, largely attributable to serotonin 2A receptor (5-HT_2A_) binding^17^. Accumulating clinical evidence suggests psilocybin, and other classical psychedelic compounds [i.e., lysergic acid diethylamide (LSD), mescaline, and dimethyltryptamine (DMT)-containing ayahuasca], possess therapeutic potential for treating psychiatric conditions including major depressive disorder^18,19^, treatment-resistant depression^20^, anxiety and depression in cancer patients^21,22^, and smoking cessation^23^. Unlike current medication options on the market, treatment response to psilocybin is rapid, observed as early as 8 h after the first dosing session^24^; robust, with medium to large between-group effect sizes according to placebo-controlled clinical trials^16,21^; and enduring, with treatment gains persisting 6 months to even 4.5 years after the last dosing session^21,22^. Based on promising results of early-stage trials, the FDA has given a breakthrough therapy designation to psilocybin for treatment-resistant depression (COMPASS Pathways) and major depressive disorder (Usona Institute).

The psychological effects of psychedelics are theorized to intervene on core domains of AUD. Within the negative emotionality domain, a growing number of studies suggest psychedelics increase positive mood and decrease negative mood and neuroticism in healthy and clinical populations^25,26^. Within the incentive salience domain, increases in the personality traits *openness* and *conscientiousness* have been observed following psilocybin treatment^26,27^. Further, in a clinical AUD sample, psilocybin reduced craving for alcohol^15^. Within the executive functioning domain, psychedelics have been shown to enhance cognitive flexibility^28^ and control^29^, mindfulness capacities^30^, and the ability to psychologically decenter from thoughts and emotions^31^. In patients with AUD, psilocybin has been shown to promote self-efficacy and improved behavioral control^32,33^. In the parent trial (NCT02061293), our team has replicated personality trait changes relevant to these domains in patients with AUD, suggesting effects on mood (decreased *neuroticism* and depression, increased positive feelings), incentive salience (decreased craving, and increased *openness* and *conscientiousness*), and executive function (increased deliberation and decreased impulsivity)^34^. Psychedelics may also enhance meta-cognition (included in the NIDA Phenotyping Assessment Battery^35^) as shifts in values and transitions from “autopilot” to “meta-aware” modes of experiential processing have been reported^36,37^. However, the neurobiological substrates of psilocybin in AUD are unknown.

Several functional MRI (fMRI) investigations have yielded preliminary insights into putative mechanisms of action in non-addicted populations. In a placebo-controlled, double-blind study in healthy controls (*n* = 38), Smigielski et al. identified functional connectivity changes in the striatum, anterior and posterior cingulate (ACC; PCC), and medial prefrontal cortex (mPFC) 2 days post-psilocybin treatment during resting state and mediation practices^37^. Decreased mPFC-PCC connectivity predicted positive mood 4 months later, potentially reflecting normalization of a circuit shown to be hyperconnected in major depressive disorder^37,38^. In treatment-resistant depression, psilocybin altered mPFC, ACC, and PCC connectivity one day post-treatment, with mPFC connectivity decreases predicting depressive symptoms 5 weeks later^39^. Findings from these post-acute studies suggest functional remodeling of key nodes of the default mode (DMN: mPFC and PCC), salience (SN: ACC and insula), and limbic (LN: striatum and amygdala) networks. Functional roles of these structures include self-referential and emotional processing, attentional and inhibitory control, and reward/motivational systems, suggesting substantial overlap with AUD neuropsychopathology.

In emotional processing paradigms, psilocybin elicited decoupling of dorsolateral PFC (dlPFC) and mPFC one day post-treatment, which predicted reductions in rumination 5 weeks later^40^. Moreover, Barrett et al. found reduced negative affect and amygdala response 7 days after psilocybin, and increased positive affect and dlPFC and mPFC responses to emotionally-conflicting stimuli^41^, pointing toward putative neural substrates of self-reported and clinical improvements in affect after psychedelics^17,42^. Findings from these neuroimaging studies utilizing negative affective paradigms can be interpreted as psilocybin-elicited downregulation of negatively valanced emotional states via prefrontal engagement of attentional and executive systems. In sum, the early clinical findings with psilocybin are promising and suggest transdiagnostic efficacy, while the few studies to date probing neurobiological mechanisms implicate core domains central to the psychopathology of AUD including negative affect, incentive salience/craving, and executive function. However, to date, there are no published neuroimaging studies investigating the effects of psilocybin in AUD or other substance use disorders.

This pilot fMRI study was conducted as part of a phase II, randomized, double-blind, placebo controlled clinical trial that investigated the efficacy of psilocybin to treat patients with AUD (NCT02061293). We sought to characterize psilocybin-induced alterations in neural reactivity to alcohol and emotional cues which may account for therapeutic effects of psilocybin in patients with AUD. Given the small sample size of this pilot, we utilized a whole-brain approach to describe psilocybin’s effects on global brain functioning. Finding from this study may serve as a springboard for future hypotheses about psilocybin’s mechanism of action for disorders of addiction.

## Methods

### Parent trial and fMRI sub-study

This study was approved by the Heffter Research Institute, the institutional review board of New York University Grossman School of Medicine, the US Food and Drug Administration and Drug Enforcement Administration, and the New York State Bureau of Narcotics Enforcement. All participants provided written, informed consent, in accordance with the Declaration of Helsinki.

The parent study methods and primary outcomes are described in detail elsewhere^16,43^. Briefly, inclusion criteria were: (1) age 25 to 65 years old, (2) confirmed AUD diagnosis using the Structured Clinical Interview for DSM-IV^44^, and (3) had at least 4 heavy drinking days in the past 30 days. Participants were excluded from the study if they had a major psychiatric or substance use disorder other than AUD; any hallucinogen use in the past year or more than 25 lifetime uses; or contraindicated medical conditions or exclusionary medications. Participants in the main trial were randomly assigned to receive two administrations of psilocybin or active placebo (diphenhydramine) with 12 weekly therapy sessions provided by two therapists. Before the medication session, all participants received 4 psychotherapy sessions featuring motivational interviewing and cognitive behavioral therapy, and educational preparation for managing and making use of the psilocybin session (see Bogenschutz and Forcehimes^43^ for further information).

A subsample of fourteen participants from the parent clinical trial consented to participate in the ancillary fMRI study and were randomized to psilocybin (*n* = 6) or placebo (*n* = 8). The timeline followback (TLFB) was used to quantify baseline drinking, yielding percent heavy drinking days (PHDD), drinks per day (DPD), and percent drinking days (PDD)^45^. The Penn Alcohol Craving Scale (PACS) was used to quantify baseline craving^46^. Baseline demographic and drinking- and fMRI-related group differences were evaluated with independent sample t-tests and Chi-squared tests. Participants completed task-based functional MRI (fMRI) with a target mean range of 2–3 days before and 1–2 days after receiving their first dose of study blinded medication, consisting of either psilocybin (25 mg/70 kg) or diphenhydramine (50 mg), administered orally during a monitored 8-h drug administration session.

### fMRI acquisition and analysis

Structural and functional MRI (fMRI) images were acquired with CBI’s Siemens Skyra scanner equipped with a 20-channel radio-frequency coil. A T1 weighted image was acquired using an MPRAGE pulse sequence in the sagittal plane with an isotropic 0.8 mm resolution, TE/TR/TI = 3.1/2400/1000 ms, and 224 slices (7 min.). fMRI images were collected in the AP direction with a multi-band gradient echo EPI sequence. Parameters were axial slices with a FOV = 248 mm, TE/TR = 29/1000 ms, 3 mm isotropic resolution, 56 slices, 955 volumes, multiband factor = 8, BW = 2770/Hz/Px, and echo spacing = 0.52 ms.

### fMRI alcohol and affective cue task

To integrate cue-elicited responses to alcohol and emotionally valenced stimuli, we employed a visual cue fMRI paradigm. Following the design of Vollstadt-Klein and colleagues^47^, participants viewed pictures of alcohol-containing beverages, and negative affective, positive affective, and neutral pictures from the *International Affective Pictures Series* (IAPS)^48^. Alcohol, neutral, negative, and positive pictures were matched for color and complexity and other potentially important confounds (i.e., presence of people) and presented in pseudorandomized order. Forty pictures were presented for each stimulus category (alcohol, neutral, negative, and positive) across 8 blocks, equaling 160 stimuli across the two 12-min runs (24-min total task time). Blocks were 20 s in duration; five pictures were presented for 4 s each. Between blocks, participants were asked to rate their craving on a scale from 1 to 5 (1 = “no craving at all” and 5 = “severe craving”) within a 10 s timeframe; 15 s of fixation ensued prior to the next block. Pre-to-post treatment changes in fMRI button box craving data was assessed using two-tailed paired t-tests.

### fMRI preprocessing

Preprocessing and analysis of fMRI data was completed in SPM12 (Wellcome Trust Centre for Neuroimaging, https://www.fil.ion.ucl.ac.uk/spm) and CONN (https://www.nitrc.org/projects/conn). Preprocessing steps included slice time correction, realignment to the mean image, co-registration to the skull-stripped T1 image, normalization to MNI space, and spatial smoothing (8 mm FWHM Gaussian kernel). Scrubbing removed functional volumes exceeding 2 mm displacement using the Artifact Detection Tools toolbox and a 128 s high-pass filter removed low-frequency drift. Whole-brain statistical analyses were performed using a general linear model with task regressors convolved with the canonical hemodynamic response function. For activation analyses, 6 realignment parameters were entered as covariates to account for motion. For functional connectivity analyses, the CONN-fMRI toolbox was used to regress out parameters for white matter (5P), CSF (5P), and realignment (12P) with first-order derivatives. Next, data were band-pass filtered (0.008 0.09) and linearly detrended. After this denoising procedure, all quality control measures were above the 95% normal histogram match, suggesting the absence of associations between quality control and functional connectivity metrics^49^: max global signal change (96.5% match, ${\overline{\text{x}}}$ = 0.02, *SD* = 0.30), mean global signal (99.1% match, ${\overline{\text{x}}}$ = 0.00, *SD* = 0.31), max motion (95.4% match, ${\overline{\text{x}}}$ = 0.03, *SD* = 0.30), and mean motion (97.7% match, ${\overline{\text{x}}}$ = − 0.02, *SD* = 0.31).

### fMRI modeling and analyses

Treatment-by-time interactions were modeled at the first and second level. At the 1^st^ level, time (post > pre) and condition (alcohol > neural; negative > neutral; and positive > neutral) were modeled. At the 2nd level, the randomized treatment assignment was modeled (psilocybin > placebo and psilocybin < placebo). For within-psilocybin group effects of time, the 1st level contrasts for psilocybin participants were entered at the 2nd level with activation specified as [1] and deactivation specified as [− 1] using the post > pre contrast.

Treatment-by-time interactions were examined for whole-brain neural activation and deactivation (blood-oxygen-level dependent (BOLD) contrast) for alcohol (alcohol > neutral), negative affective (negative > neutral) and positive affective (positive > neutral) cue reactivity tasks (*p*-uncorrected < 0.005, k = 10). Significant interactions were followed up with within-psilocybin group changes (pre- to post-treatment) to determine brain regions driving the interactions (*p*-uncorrected < 0.005, k = 10).

Brain regions showing significant treatment-by-time interactions in the alcohol contrast were entered into a seed-based region of interest (ROI) using a generalized psychophysiological interactions (gPPI) approach to identify functional connectivity alterations specific to alcohol processing after controlling for the positive, negative, and neutral conditions (*p*-FWE < 0.05). For the functional connectivity analyses, gPPI modeled the entire experimental session by calculating regressor and PPI terms for each condition and generating beta weights for interaction terms (Y = Alc + Neg + Neut + Pos + ROI + Alc*ROI + Neut*ROI + Neg*ROI + Pos*ROI + error)^60^. This method enables the isolation of condition-specific modulation of connectivity.

The rationale for using a whole-brain, uncorrected *p* < 0.005 threshold—rather than an ROI FWE/FDR corrected approach—was on the basis of the following: (1) the present study’s sample size was not adequate for multiple comparison correction; (2) the absence of fMRI studies of psilocybin in alcohol use disorder (and all other substance use disorders) and cue-reactivity tasks precluded justifiable hypotheses; (3) widespread abnormalities in neural co-activation in AUD result in a large number of potential ROIs; (4) there is evidence that psychedelics alter global brain dynamics^50^; and (5) psychedelics cannot be assumed to have effects similar to traditional pharmacotherapies.

## Results

### Demographics

Two participant did not complete both study visits and fMRI malfunctioning resulted in incomplete data collection for one participant at the pre-intervention visit, resulting in the exclusion of three participants from the analysis. Thus, the final sample comprised eleven participants (psilocybin *n* = 5; placebo *n* = 6). No group differences were detected in biological sex, age, weight, baseline craving, baseline percent heavy drinking days, baseline drinks per day, or pre/post fMRI framewise displacement (Table 1). However, the psilocybin group scored significantly higher in percent drinking days at baseline relative to the placebo group (Table 1). fMRI scans were collected on average 2.55 days before psilocybin treatment (*SD* = 1.75; range 1–6) and 1.45 days after treatment (*SD* = 0.68, range 1–3), falling within the mean target range of 2–3 days before and 1–2 days after. No group differences were detected in the number of days between the first fMRI and treatment (*t*[9] = -0.77, *p* = 0.462), between treatment and the second fMRI (*t*[9] = 0.229, *p* = 0.82), or between the first fMRI and the second fMRI (*t*[9] = -0.571,* p* = 0.582).

**Table 1:** Participant demographics and baseline alcohol craving and consumption.

	Psilocybin	Placebo	Baseline group differences
Sample size (male/female)	5 (4/1)	6 (3/3)	χ^2^(1) = 1.06; p = 0.30
Race/ethnicity	White (4) Hispanic (1)	White (4) Hispanic (1) Black (1)	χ^2^(2) = 0.917; p = 0.63
Annual income	$97,500 ($54,237) Range: 25–150 K	$148,333 ($109,025) Range: 50–360 K	t(8) = 0.85, p = 0.42
Age, years	48.80 (10.52) Range: 35–63	44.00 (12.62) Range: 27–59	t(9) = − 0.68; p = 0.52
Weight (lbs)	201.00 (34.70) Range: 160–256	159.67 (49.36) Range: 112–246	t(9) = − 1.57; p = 0.15
Craving^a^	16.00 (6.44) Range: 8–23	14.67 (3.72) Range: 8–19	t(9) = − 0.43; p = 0.68
PHDD^b^	17.86 (17.50) Range: 0–39.29	14.29 (17.35) Range: 0–42.86	t(9) = − 0.34; p = 0.74
DPD^b^	2.58 (0.73) Range: 1.37–3.30	1.31 (1.64) Range: 0–4.18	t(9) = − 1.60; p = 0.15
PDD^b^	77.14 (17.05) Range: 60.71–96.43	29.17 (33.61) Range: 0–89.29	t(9) = − 2.88; p = 0.02*
fMRI FD	0.13 (0.03) Range: 0.10–0.18	0.12 (0.02) Range: 0.09–0.13	t(9) = − 1.14; p = 0.28

Reported as means, (standard deviations), and ranges; ^a^Penn Alcohol Craving Scale; ^b^Timeline Follow Back (TLFB); PHDD: percent heavy drinking days; DPD: drinks per day; PDD: percent drinking days; FD: framewise displacement *p < 0.05.

### Alcohol cue neural reactivity

**Figure 1:**
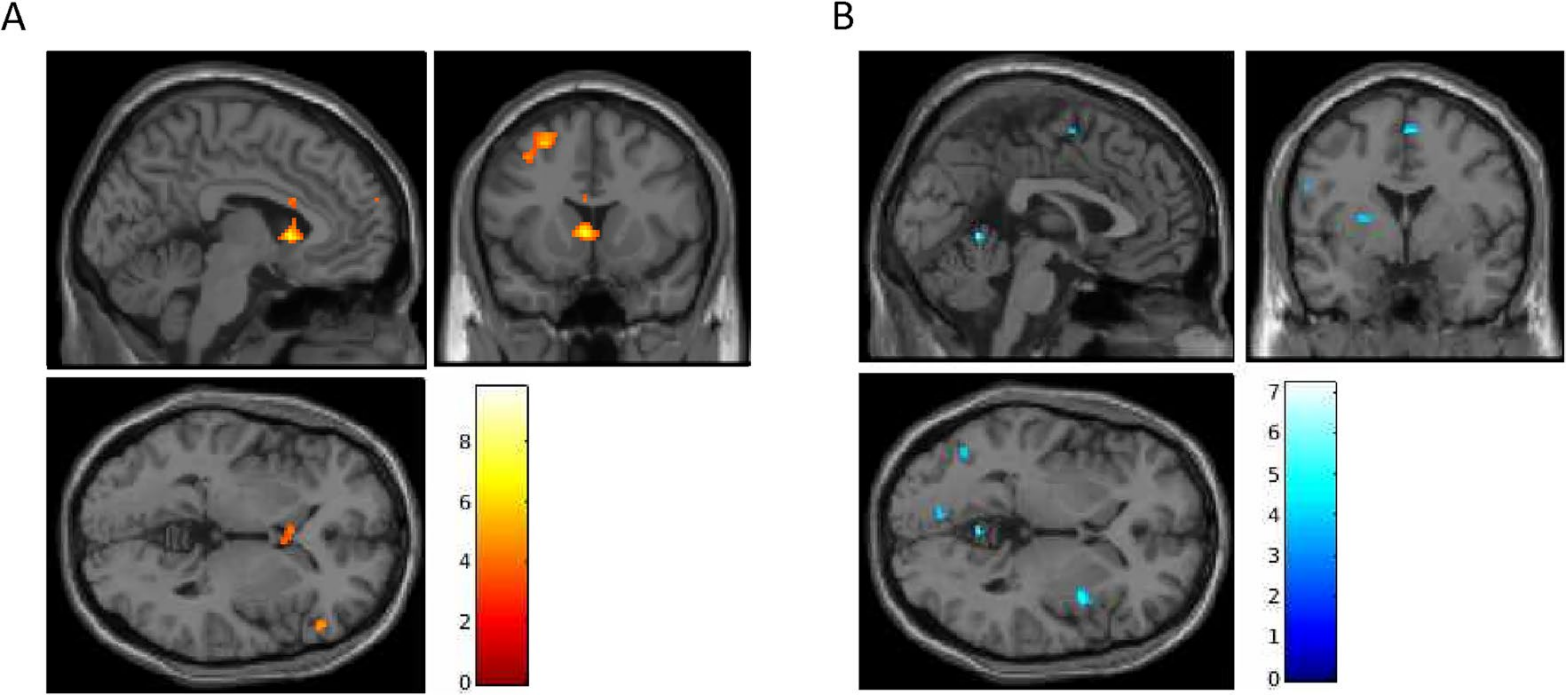
Pre-to-post treatment interactions in BOLD signal during alcohol cue processing (alcohol > neutral; *p* < 0.005; *t*-statistics). (**A**) Post > Pre. (**B**) Pre > Post.

On the alcohol cue reactivity task, treatment-by-time interactions detected increased activation in 8 clusters (Fig. 1A, Table 2). Of these, 6 clusters showed within-psilocybin treatment effects, including: left superior medial prefrontal cortex (mPFC), right ventrolateral PFC (vlPFC = inferior frontal gyrus [IFG]), left dorsolateral PFC (dlPFC = middle frontal gyrus [MFG]), and bilateral caudate (Table 2). Deactivation treatment-by-time interactions were detected in 17 clusters (Fig. 1B, Table 2). Of these, 8 clusters showed within-psilocybin treatment effects, including: right insula, motor areas (right supplementary motor area [SMA] and left precentral gyrus [PreCG]), cerebellum (vermis 4/5), and left lingual, left superior occipital (SOG), and left middle temporal (MTG) gyri (Table 2).

**Table 2:** Alcohol cue-reactivity: treatment-by-time interaction and within-psilocybin effect of time.

Alcohol cue		Treatment by time interaction				Psilocybin main effect of time			
	ROI	MNI coordinates	Voxels	Cluster p-unc	Peak p-unc	MNI coordinates	Voxels	Cluster p-unc	Peak p-unc
Activation	R IFG triangularis	48, 30, 8	56	0.067^#^	** < 0.001**	56, 30, 4	11	0.216	**0.001**
	L caudate	− 4, 14, 4	168	0.004**	** < 0.001**	− 10, 12, 4	64	0.007*	** < 0.001**
	L MFG	− 26, 16, 54	188	0.002**	** < 0.001**	− 38, 18, 50	123	< 0.001**	** < 0.001**
	L MFG	− 38, 38, 30	47	0.090^#^	** < 0.001**	− 38, 38, 30	22	0.088^#^	**0.001**
	L dorsal mPFC	− 6, 64, 24	11	0.401	**0.001**	− 2, 44, 34	145	< 0.001**	** < 0.001**
	R caudate	22, 22, 4	11	0.401	**0.001**	16, 24, − 2	30	0.050*	** < 0.001**
	R MFG	30, − 2, 54	11	0.401	**0.001**				
	L superior TP	− 42, 4, − 16	17	0.295	** < 0.001**				
Deactivation	vermis 4/5	− 2, − 54, − 2	23	0.224	** < 0.001**	6, − 56, − 8	13	0.180	** < 0.001**
	L cerebellar tonsil	− 26, − 46, − 40	26	0.197	** < 0.001**	− 22, − 46, − 49	39	0.028*	** < 0.001**
	R SMA	8, − 2, 60	34	0.144	** < 0.001**	2, − 10, 54	14	0.166	** < 0.001**
	L MTG	− 56, − 34, 4	22	0.234	** < 0.001**	− 50, − 54, − 4	97	0.001**	** < 0.001**
	L SOG	− 14, − 92, 26	33	0.149	**0.001**	− 16, − 90, 28	24	0.166	** < 0.001**
	R insula	40, 6, 0	42	0.107	** < 0.001**	38, 6, 6	46	0.019*	** < 0.001**
	L lingual gyrus	− 10, − 78, − 2	28	0.182	**0.001**	− 20, − 54, − 4	24	0.076^#^	** < 0.001**
	L precentral gyrus	− 56, 2, 26	11	0.401	**0.003**	− 46, 6, 42	19	0.110	**0.001**
	L SFG	− 20, 38, 28	26	0.197	** < 0.001**				
	R IFG operculum	50, 18, 8	14	0.342	** < 0.001**				
	R cerebelum 9	8, − 62, − 54	17	0.295	** < 0.001**				
	L MOG	− 42, − 64, 4	56	0.067^#^	** < 0.001**				
	L RO/insula	− 42, − 18, 20	10	0.424	** < 0.001**				
	R cuneus	18, − 64, 36	13	0.360	**0.001**				
	L putamen	− 26, 2, 10	29	0.175	**0.001**				
	R cerebellum 9	18, − 48, − 52	31	0.161	**0.001**				
	R OFC	20, 44, − 18	29	0.175	**0.001**				

L: left; R: right; IFG: inferior frontal gyrus; SFG: superior frontal gyrus; MFG: middle frontal gyrus; TP: temporal pole; MTG: middle temporal gyrus; MOG: middle occipital gyrus; SOG: superior occipital gyrus, RO: rolandic operculum; peak *p*-uncorrected < 0.005; cluster level significance: ^#^< 0.1, *< 0.05, **< 0.005. Significant values are in bold (p < 0.005).

### Negative emotional cue neural reactivity

**Figure 2:**
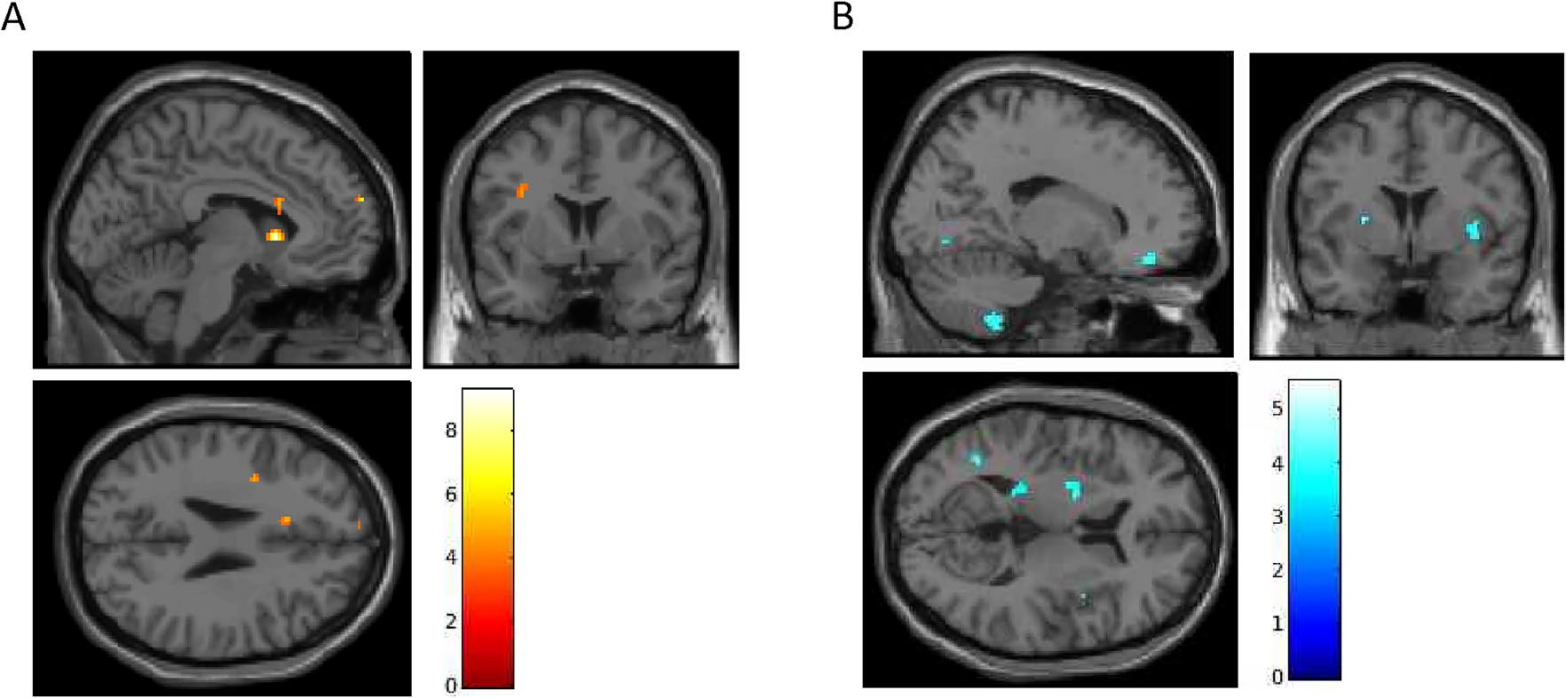
Pre-to-post treatment interactions in BOLD signal during negative cue processing (negative > neutral; *p* < 0.005; *t*-statistics). (**A**) Post > Pre. (**B**) Pre > Post.

In the negative affective cue task, treatment-by-time interactions detected increased activation in 5 clusters (Fig. 2A, Table 3). Of these, 3 clusters showed within-psilocybin treatment effects, mirroring areas from the alcohol cue reactivity task, including the left caudate, left mPFC, and left dlPFC, and uniquely, the right supramarginal gyrus (SMG; Table 3). Deactivation treatment-by-time interactions were detected in 13 clusters (Fig. 2B, Table 3). Of which, 6 clusters showed within-psilocybin treatment effects, including the right insula, left MTG, bilateral lingual gyri, and cerebellum (left 4/5 and right 9; Table 3).

**Table 3:** Negative cue-reactivity: treatment-by-time interaction and within-psilocybin effect of time.

Neg. affect cue		Treatment by time interaction				Psilocybin main effect of time			
	ROI	MNI coordinates	Voxels	Cluster p-unc	Peak p-unc	MNI coordinates	Voxels	Cluster p-unc	Peak p-unc
Activation	L caudate	− 6, 15, 2	161	0.005**	** < 0.001**	− 4, 14, 2	99	0.021*	** < 0.001**
	L dmPFC	− 8, 64, 24	29	0.187	** < 0.001**	− 8, 64, 24	18	0.186	** < 0.001**
	R SMG	40, − 34, 38	21	0.258	** < 0.001**	36, − 40, − 16	10	0.261	**0.001**
	R IFG triangularis	48, 32, 6	19	0.282	**0.001**				
	R parahippocampus	30, − 22, − 22	29	0.202	**0.002**				
Deactivation	R cerebellum 9	8, − 62, − 54	12	0.394	** < 0.001**	18, − 48, − 54	13	0.202	**0.001**
	R lingual gyrus	22, − 72, − 8	10	0.438	**0.001**	18, − 66, − 6	29	0.065^#^	** < 0.001**
	L lingual gyrus	− 8, − 76, − 2	11	0.415	**0.002**	− 20, − 54, − 6	40	0.034*	** < 0.001**
	L cerebellum 4/5	− 20, − 46, − 28	12	0.394	** < 0.001**	− 18, − 46, − 28	43	0.029*	** < 0.001**
	R insula	42, 4, 8	67	0.053^#^	** < 0.001**	44, 8, − 6	119	0.001*	** < 0.001**
	L MTG	− 40, − 58, 10	14	0.356	**0.001**	− 48, − 56, 2	14	0.186	**0.002**
	L SOG	− 14, − 92, 26	13	0.374	**0.001**				
	L RO/insula	− 42, − 18, 22	10	0.438	**0.001**				
	R cerebellum 9	18, − 44, − 54	37	0.139	**0.001**				
	L hippocampus	− 22, − 36, 8	25	0.219	**0.001**				
	L putamen	− 22, − 2, 8	52	0.084^#^	**0.001**				
	R rectus	14, 42, − 16	46	0.102	** < 0.001**				
	R SMA	8, − 6, 60	35	0.149	** < 0.001**				

L: left; R: right; dmPFC: dorsomedial prefrontal cortex; SMG: supramarginal gyrus; IFG: inferior frontal gyrus; MTG: middle temporal gyrus; SOG: superior occipital gyrus; RO: rolandic operculum; SMA: supplementary motor area; peak *p*-uncorrected < 0.005; cluster level significance: ^#^< 0.1, *< 0.05, **< 0.005. Significant values are in bold (*p* < 0.005).

### Positive emotional cue neural reactivity

**Figure 3:**
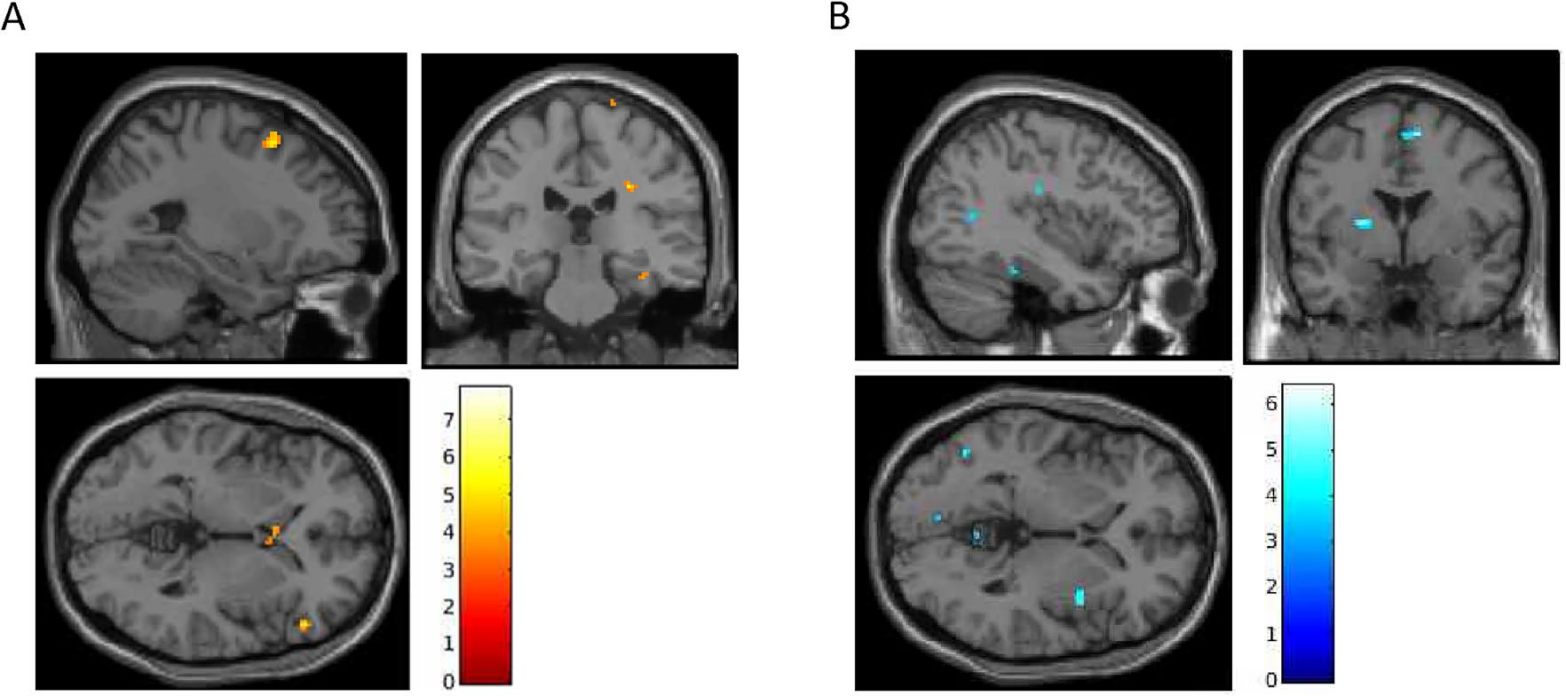
Pre-to-post treatment interactions in BOLD signal during positive cue processing (positive > neutral; *p* < 0.005; *t*-statistics). (**A**) Post > Pre. (**B**) Pre > Post.

In the positive affective cue task, treatment-by-time interactions detected increased activation in 7 clusters (Fig. 3A, Table 4). Of these, 4 clusters showed within-psilocybin treatment effects, including the left mPFC, left vlPFC, and right hippocampus (Table 4). Deactivation interactions were identified in 20 clusters (Fig. 3B, Table 4). Of which, 9 clusters showed within-psilocybin treatment effects, including the left hippocampus, right SMA, left MTG, left SOG, and cerebellum (vermis 4/5, right 8/9; Table 4).

**Table 4:** Positive cue-reactivity: treatment-by-time interaction and within-psilocybin effect of time.

Pos. affect cue		Treatment by time interaction				Psilocybin main effect of time			
	ROI	MNI coordinates	Voxels	Cluster p-unc	Peak p-unc	MNI coordinates	Voxels	Cluster p-unc	Peak p-unc
Activation	L MFG	− 26, 14, 58	128	0.010*	** < 0.001**	− 34, 12, 52	112	0.001**	** < 0.001**
	L dmPFC	− 6, 64, 24	15	0.329	** < 0.001**	− 8, 50, 34	154	< 0.001**	** < 0.001**
	L MFG	− 38, 40, 30	10	0.428	** < 0.001**	− 36, 56, 2	22	0.250	** < 0.001**
	R hippocampus	28, − 18, − 18	16	0.313	**0.001**	28, − 20, − 22	13	0.191	** < 0.001**
	R IFG triangularis	50, 34, 6	62	0.057^#^	** < 0.001**				
	L caudate	− 4, 14, 4	145	0.006*	** < 0.001**				
	R hippocampus	38, − 22, − 18	12	0.383	**0.002**				
Deactivation	L hippocampus	− 20, − 40, 6	24	0.218	** < 0.001**	− 20, − 40, 6	13	0.191	** < 0.001**
	R SMA	10, − 2, 60	46	0.096^#^	** < 0.001**	6, − 8, 64	18	0.128	**0.001**
	vermis 4/5	6, − 58, − 12	25	0.289	** < 0.001**	4, − 54, − 8	12	0.209	**0.001**
	L SOG	− 14, − 92, 26	25	0.289	** < 0.001**	− 16, − 94, 26	35	0.041*	** < 0.001**
	L MTG	− 46, − 62, 0	12	0.383	**0.001**	− 44, − 54, − 4	38	0.034*	** < 0.001**
	R SMA	12, 24, 58	10	0.428	**0.001**	12, 0, 62	11	0.228	** < 0.001**
	L MTG	− 40, − 58, 10	14	0.346	**0.001**	− 44, − 54, − 4	99	0.002**	** < 0.001**
	vermis 4/5	0, − 54, − 2	10	0.428	**0.001**	4, − 54, − 8	12	0.209	** < 0.001**
	R cerebellum 8/9	20, − 48, − 50	38	0.127	**0.001**	18, − 50, − 54	17	0.138	< 0.001
	R MFG	34, 46, 8	30	0.171	** < 0.001**				
	L putamen	− 26, 2, 8	35	0.141	** < 0.001**				
	L SMA	− 10, 4, 56	17	0.298	** < 0.001**				
	L fusiform	− 34, − 30, − 24	14	0.346	** < 0.001**				
	R cerebellum 9	6, − 62, − 52	18	0.285	** < 0.001**				
	L RO/insula	− 42, − 18, 22	20	0.260	**0.001**				
	L insula	− 30, 16, − 18	11	0.405	**0.001**				
	R cuneous	20, − 64, 36	11	0.405	**0.001**				
	R OFC	2, 46, − 14	24	0.218	**0.001**				
	L SFG	− 18, 38, 28	23	0.228	**0.001**				
	L lingual gyrus	− 8, − 78, − 2	13	0.364	**0.002**				

L: left, R: right; MFG: middle frontal gyrus, dmPFC: dorsomedial prefrontal cortex, IFG: inferior frontal gyrus, SMA: supplementary motor area, SOG: superior occipital gyrus, MTG: middle temporal gyrus, RO: rolandic operculum; OFC: orbital frontal cortex, SFG: superior frontal gyrus; peak *p*-uncorrected < 0.005; cluster level significance: ^#^< 0.1, *< 0.05, **< 0.005. Significant values are in bold (*p* < 0.005).

### Functional connectivity

Based on activation findings showing (i) significant treatment-by-time interactions and (ii) significant within-psilocybin effects of time for the alcohol contrast, the following regions were run in a seed-based gPPI functional connectivity analysis: (1) left caudate, (2) right caudate, (3) right inferior frontal gyrus (vlPFC), (4) left middle frontal gyrus (dlPFC), and (5) left superior medial frontal cortex (mPFC). With a *p*-FWE threshold of < 0.05, results revealed increased functional connectivity between (1) left caudate and anterior cingulate cortex (ACC; *p*-FWE = 0.040) and (2) right inferior frontal gyrus (IFG) pars triangularis and right precentral gyrus/dlPFC (*p*-FWE = 0.016) for the alcohol contrast. However, no connectivity changes in the right caudate or mPFC were detected (*p*-FWE < 0.05). No decreases in functional connectivity (pre > post) were detected across ROIs.

### Craving data

fMRI button box malfunction resulted in incomplete data collection of craving ratings (final sample: psilocybin *n* = 4; placebo *n* = 1). Paired sample t-test revealed a significant decrease in craving across all cue types in the psilocybin group (t[3] = 5.568, p = 0.0114). Individual comparisons showed no significant pre-to-post change in the psilocybin group for: alcohol cues (t[3] = 2.718, p = 0.0727), positive cues (t[3] = 1.528, p = 0.2241), negative cues (t[3] = 2.050, p = 0.1327), or neutral cues (t[3] = 1.321, p = 0.2783).

## Discussion

The present study sought to characterize psilocybin-induced alterations in neural activity to alcohol and emotional cues which may account for therapeutic effects in patients with alcohol use disorder (AUD). Psilocybin treatment was associated with engagement of various prefrontal cortical areas (lateral and medial PFC) and the caudate, and disengagement of the insula, motor and cerebellar areas, and temporal, parietal, and occipital cortices. These post-acute effects (i.e. occurring in the days following psilocybin administration) largely implicate brain areas previously reported to be acutely affected by psilocybin^51^. Importantly, group-by-time interactions were mostly driven by changes in the psilocybin group, suggesting that psilocybin-assisted therapy alters neural activity across the cortex and within multiple limbic structures. The high prevalence of overlapping regions across conditions suggests treatment effects were largely non-specific to stimulus type (alcohol, negative, and positive cues), and possibly reflects alterations to the saliency of visual stimuli, affective processing, or a general mood stabilizing effect.

Psilocybin-treated patients displayed increased caudate, mPFC, vlPFC, and dlPFC engagement across multiple cue types, suggesting functional reorganization of structures involved in emotional regulation, response inhibition, goal-directed action^47^, and executive functioning^5^. However, the directionality of some of the effects are—at initial pass—inconsistent with normalization of AUD-related dysfunction as meta-analyses indicate hyperactive frontostriatal circuits in AUD. Specifically, studies have reported hyperactivity of the mPFC and dorsal striatum in response to alcohol cues, relative to healthy controls, and treatment-induced downregulation of this pathway within AUD samples^51,52^. While this warrants caution when interpreting the present study findings, a few lines of evidence offer potential explanations.

First, hyperactivity to alcohol cues in these regions are frequently reported in the context of *hypoactive* responses to other stimulus categories (i.e., negative/stress, neutral, positive stimuli)^53,54^. Such alcohol-specific hyperactivity supports the notions of pathological incentive salience toward alcohol cues, and concomitant devaluation of non-drug stimuli in AUD^7^. Therefore, it is plausible that increased activity in these brain regions across alcohol and affective stimuli reflects a broadening of incentive salience and changes in general affective processing. Such a widening of the attentional scope may be critical to belief updating in predictive coding and Bayesian models of addiction^55,56^, as has been posited to be a mechanism of action of psychedelics^57^.

Secondly, directionality has been mixed as studies have also reported hypoactivity within frontostriatal regions in AUD. For example, hypoactive mPFC and striatum responses to alcohol and negative/stress images, in contrast to hyperactive responses in these regions to neutral/relaxing images, have been reported in AUD compared to healthy controls^58,59^. Since both hyper- and hypoactivity in the mPFC predicted drinking behavior and relapse in these studies, valence-dependent responses in the mPFC may be clinically relevant. Notably, we observed decreases in orbitofrontal cortex (OFC), a subregion of the vmPFC, and increases in the dmPFC, areas responsible for emotional and cognitive aspects of self-referential processing, respectively^60^. In line with our findings, successful inhibition of cue-induced cocaine craving has been negatively associated with OFC activity and positively associated with vlPFC activity in the right hemisphere^61^. Thus, we speculate psilocybin might dampen the emotional and enhance the cognitive self-relevancy of emotionally charged stimuli. It is also important to consider that mPFC and caudate were activated in concert with ventral and dorsal divisions of the lateral PFC, matching what is observed in healthy controls who show greater lateral PFC recruitment compared to AUD patients^62^. Additionally, greater medial and lateral PFC activity during the regulation of alcohol craving and negative emotions has been observed in patients with AUD^63^. Thus, while psilocybin-induced increases in medial PFC is inconsistent with normalization of alcohol cue sensitization in AUD^52^, patterns match neural signatures of cognitive regulation, suggesting that psilocybin may enhance top-down executive control, rather than blunt the saliency of alcohol-related cues^64^. Future studies should consider the complex and potentially opposing roles of ventral, dorsal, and orbital divisions of the medial PFC, and contemporaneous lateral PFC co-activation, when evaluating psilocybin modulating effects on cue-reactivity.

Further support for psilocybin’s putative effects on cognitive regulation can be drawn from the neurobiological underpinnings of attentional and inhibitory control in AUD. For example, IFG response is negatively associated with attentional biases to drug cues^65^; heightened dlPFC and vmPFC is observed during alcohol interference in a Go-NoGo task^66^; diminished dlPFC recruitment is observed when making reward-related decisions and processing negative prediction errors^67^; and dlPFC stimulation reduces alcohol craving^68^. In the context of psilocybin treatment, one study found increased dlPFC, vlPFC, and mPFC response in an emotional conflict Stroop task^41^, and another found mPFC functional connectivity changes during a focused attention meditation practice^37^. Considering this research in the context of AUD suggests that psilocybin might diminish preference for alcohol cues and engage hubs of inhibitory control. However, follow-up studies using executive functioning tasks are needed to directly test this proposition.

While comparisons with other studies of psilocybin’s action are difficult due to heterogeneities in clinical samples, assessment time points (acute versus post-acute), and task designs, there has been some consistency in reported brain regions, including: the mPFC, a hub of the DMN (see Gattuso et al.^69^ for a review of psychedelic effects on the DMN), the ACC and insula, nodes of the SN, and lateral PFC, a hub of the executive control network. Focusing strictly on post-acute effects, psilocybin has been shown to induce connectivity changes in the cingulum, striatum, and mPFC, with decreased mPFC-PCC connectivity predictive of positive mood 4 months later among health controls^37^. In treatment-resistant depression, psilocybin altered mPFC, ACC, and PCC connectivity one day post-treatment, with decreases in mPFC connectivity predicting depressive symptoms 5 weeks later^39^. In a negative affective task similar to the one employed in the present study, dlPFC and mPFC decoupling with the amygdala one day post-psilocybin has been shown to predict reductions in rumination 5 weeks post-treatment^40^. Moreover, Barrett and colleagues found psilocybin increased positive affect and increased PFC response to emotionally conflicting stimuli^41^.

While we did not observe functional connectivity changes in the mPFC as has been reported in other samples, we found increases in ACC-caudate and vlPFC-precentral gyrus connectivity, suggesting psilocybin may modulate frontostriatal and motor circuits, respectively. Whether these changes reflect top-down or bottom-up modulation deserves attention in future studies using effective connectivity approaches. Our findings of increased PFC activity and functional connectivity with striatal and motor areas add to this growing body of literature, and together, independent research groups are beginning to converge on putative therapeutic substrates of psychedelics^17,37, 41^.

Augmented striatal activity to alcohol cues has been most widely reported in the ventral striatum (nucleus accumbens^53^) and putamen, responsible for reward/motivation and motor control/habitual behavior, respectively, whereas the caudate appears to contribute more to goal-directed action and cognitive control^70^. Given this functional distinction (and concomitant PFC activation), heightened caudate response and caudate-ACC connectivity post-treatment might reflect top-down cognitive control and diminished emotional perturbation. Relatedly, diminished functional connectivity between the striatum and ACC has been associated with AUD severity in a response inhibition task^71^, and abstainers display stronger striatal-ACC connectivity than non-abstainers^72^. Intriguingly, we did not observe decreases in the nucleus accumbens or amygdala as expected. Decreases in the left putamen were evident in the interaction but nonsignificant for within-psilocybin comparisons. Acute reductions in left putamen have been reported following psilocybin administration^51^. In light of these considerations, we speculate that the effects observed in the present study reflect a state of improved self-regulatory control in relation to long-term goal pursuit (sobriety or reduced drinking) and emotional equipoise irrespective of changing environmental stimuli^63^.

Psilocybin-treated patients also displayed broad reductions in insular, motor, temporal, occipital, and cerebellar activity relative to placebo controls. These findings are in line with an activation likelihood estimation meta-analysis in AUD that found hyperactivity and treatment-induced reductions in these brain regions, including after cue-exposure therapy^52,72,73^. Overall, the patterns of deactivation observed after psilocybin point toward normalization. For example, greater activation in insular, temporal, parietal, and occipital cortices have generally been found during alcohol cues exposure in AUD versus health controls^61,68,71^ (with some inconsistencies^52^). A role for the cerebellum in addiction and craving has also emerged^74^, with activity positively correlating with AUD severity^6^. Our findings of attenuated cerebellar response support a growing consensus of its contributions to higher-order cognitive functions such as negative emotionality, salience detection, executive control, memory, and self-reflection^75^. Acutely, psilocybin has also been shown to decrease activity in the insula, hippocampus, motor cortex, and temporal areas, although directionality might be dependent on relative versus absolute measurement^51^. Psychedelics modulate areas rich in 5-HT1A receptor expression, such as the insula, raising the possibility that psilocybin may exert inhibitory effects on the insula via agonism at 5-HT1A receptors^76^. In relation to AUD, decreases in insular activity are in line with previous work showing insular hyperactivity and treatment-induced reductions in AUD^52^. The insula has long been associated with interoceptive components of craving and negative affect^77^. Psilocybin-specific decreases in insular activity were robust for alcohol and negative affective contrasts, but not for positive affective cues, suggesting that attenuation of interoceptive processing is specific to craving and negative affect states.

Unique to positive affective cues, psilocybin reduced left and increased right hippocampus engagement. Interestingly, hemispheric asymmetries have been established for emotional processing, with left hippocampal lateralization occurring when viewing negative versus neutral pictures^78^. Others have observed increases in relative cerebral blood flow in the right hippocampus acutely after psilocybin administration^51^, raising the question whether these changes persist or undergo temporal reconfiguration that ultimately results in durable clinical effects. We speculate that these lateralized, affect-specific responses might reflect the facilitation of natural, non-drug rewards regaining reinforcing properties and a resetting of the hedonic set point as has been qualitatively reported in the parent study^79^.

Recent developments in establishing a neural signature of craving have included temporal, parietal, occipital, and cerebellar regions, expanding the neurobiology of addictions beyond the confines of the mesocorticolimbic circuitry which has dominated the field’s focus^80^. Koban and colleagues posit that co-activation of visual and posterior attentional areas may be critical to ascribe personal meaning to rudimentary percepts^80^, as has been established for complex emotional states—such as fear and sadness—which are highly embedded in the visual system^81^. From this perspective, it is possible that personal associations with alcohol and emotional contexts are attenuated though PFC engagement and contemporaneous posterior disengagement, giving rise to a decentered, nonjudgmental, and nonreactive perspective as has been reported in the early stages of mindfulness meditation interventions^82,83^. However, in the absence of brain-behavior analyses and relevant fMRI paradigms, extreme caution is warranted when inferring the cognitive and psychological processes underlying these brain findings. Well-powered studies are needed to examine the relationships between these neural correlates and the proposed cognitive constructs.

## Limitations

This study has major limitations worth noting. The single most limiting factor is the small sample size which restricts generalizations and limits statistical power. Rather than approaching this small dataset with ROI hypotheses, we chose to report whole-brain level changes to serve as a foundation for other work to replicate or disconfirm. We utilized a balanced statistical thresholding approach and sought to isolate psilocybin-specific effects by focusing on treatment-by-time interactions that demonstrated within-psilocybin effects of time. While this approach provides an unbiased method to explore these data in the absence of previous studies, it results in an elevated type 1 error rate due to multiple comparisons. Another limitation related to the small sample size is the lack of control for variables which may contribute to BOLD response in cue-reactivity designs (due to the need to conserve degrees of freedom). For example, biological sex, smoking status, and age may influence AUD responses to cues. The within-subject design of the study, inclusion of motion parameters as covariates, and absence of baseline between-group differences, partially mitigates this concern, but these factors should be accounted for in better powered studies. Other limitations include a nondiverse, homogeneous population which was primarily Caucasian, of young adult age, and of middle-to-high socioeconomic status. Studies with diverse samples are critical to determining for whom psilocybin treatment is (most) beneficial and if shared neural mechanisms underpin therapeutic improvements across populations. All of the findings of this pilot study require replication in larger and more diverse samples before they can be accepted as generalizable.

## Conclusion

In summary, this randomized, controlled pilot study provides the first data on neurobiological changes occasioned by psilocybin-assisted therapy in patients with AUD. Key findings are: (1) increased engagement of frontal circuits; (2) widespread disengagement of temporal, parietal, occipital, and cerebellar brain regions; and (3) consistently overlapping neurobiological circuits across stimulus categories, suggestive of alterations to affective processing. While caution is urged due to sample size and lack of stringent multiple comparison correction, the findings are encouraging, suggest large effect sizes, and reveal potential therapeutic neural changes attributable to psilocybin in AUD.

Promisingly, if fMRI metrics prove to be strong proxies of the purported rapid, robust and enduring salutary effects of psilocybin, future investigation in this area holds potential to (i) elucidate the etiology of AUD (ii) identify novel neural targets seeking to optimize and sustain treatment gains (i.e. using neurostimulation technologies or non-psychedelic 5-HT_2A_ agonists), (iii) reveal transdiagnostic mechanisms of psychiatric conditions, and (iii) facilitate precision-based medicine for AUD and other disorders of addiction.

## Data Availability

The datasets used and analyzed are available from the corresponding author on reasonable request.
